# Impact of the *TTN* C > T Polymorphism on Selected Variables of Aerobic and Anaerobic Capacity after a 12-Week Training Program

**DOI:** 10.5114/jhk/191847

**Published:** 2024-09-26

**Authors:** Agata Leońska-Duniec, Ewelina Maculewicz, Myosotis Massidda, Maciej Buryta, Andrzej Mastalerz, Paweł Cięszczyk

**Affiliations:** 1 Faculty of Physical Education, Gdansk University of Physical Education and Sport, Gdansk, Poland.; 2 Faculty of Physical Education, Jozef Pilsudski University of Physical Education in Warsaw, Warsaw, Poland.; 3 Department of Medical Sciences and Public Health, University of Cagliari, Cagliari, Italy.; 4 Institute of Physical Culture Sciences, University of Szczecin, Szczecin, Poland.

**Keywords:** physical activity, exercise metabolism, titin, sport genetics, Wingate test

## Abstract

The TTN gene encodes a large muscle protein called titin, which provides structure, stability, and flexibility to skeletal and cardiac sarcomeres. The aim of this study was to determine whether the TTN C > T polymorphism (rs10497520) influenced training-induced changes in selected variables of aerobic and anaerobic capacity. We studied genotypes distribution in a group of 156 Caucasian females examined for aerobic capacity evaluated by maximal oxygen uptake (VO_2max_), and anaerobic capacity measured with the Wingate anaerobic test, before and after a 12-week training program. The most important finding was a genotype by training interaction for anaerobic capacity (AnC) during the Wingate test (p = 0.003). In response to training, carriers of the CT and TT genotypes demonstrated a significant increase in the total amount of work accomplished. We also showed that the applied training program improved all the Wingate test variables in the CT + TT genotype group by 10%. The obtained results suggest that the CT and TT genotypes may enhance anaerobic power and anaerobic capacity changes induced by regular training. We also suggest that T allele carriers may possess a metabolic adaptive advantage towards the anaerobic metabolism. Thus, the TTN gene may be considered a promising marker used in sports science, underlying variability in achieving sporting goals in events where the anaerobic energy system predominates.

## Introduction

Regular physical activity affords many physiological and psychological health benefits. The most important being the body’s training responses concerning muscle tissue functions (Becker et al., 2001). However, some individuals exhibit variable responses to comparable training volume and intensity. Recently, it was demonstrated that people with the same genotypes present similar exercise responses contrary to those with different genotypes, suggesting that genetic factors play a significant role in determining individual variability in response to physical activities (Leońska-Duniec et al., 2016; Cholewa et al., 2016; Williams et al., 2017; Maciejewska-Skrendo et al., 2019). Genetic-related trainability may be related to positive responses, no response or even negative responses to specific training programs (Petr et al., 2018). One of the most promising genetic markers for adaptation of skeletal and cardiac function to training is the gene encoding titin (*TTN*; 2q31.2) (Rankinen et al., 2004).

Titin is the third most abundant component in both skeletal and cardiac sarcomeres (Maruyama, 1976; Wang et al., 1979). Four functionally and structurally different regions with frequent sequence domain repeats can be characterized: an amino-terminal Z-disc, a middle I-band and A-band parts, as well as a terminal part with a free carboxyl group that spans the M-line (Chauveau et al., 2014; Leońska-Duniec and Maciejewska-Skrendo, 2021). This protein is critically important for maintaining sarcomere integrity, development of passive tension and elasticity in myocytes, regulation of active muscle contraction, and intracellular signaling (Linke, 2018; Monroy et al., 2012). Therefore, *TTN* polymorphisms which affect the properties of titin filaments might regulate some muscle properties linked to elite athlete status (Rankinen et al., 2004; Stebbings et al., 2018). It should be noticed that carrying specific alleles provides a partial advantage to achieving outstanding sports performance, although if they are missing, it does not prevent from achieving elite status (Petr et al., 2019).

The *TTN* polymorphisms have been mainly linked to disorders of cardiac and skeletal muscle (Chauveau et al., 2014; Lewinter and Granzier, 2013). However, their connection with athletic performance, post-training adaptations, athlete status or injury risk is almost unknown. The best described *TTN* polymorphic site in the context of sports predisposition is the C > T (Lys1201Glu, rs10497520) polymorphism, which causes an amino acid change from lysine to glutamic acid. This substitution might influence, either favorably or unfavorably, properties of skeletal and cardiac muscles and in general, elite performance in sport (Stebbings et al., 2018). As evidence, this variation has been associated with changes in maximal oxygen uptake (VO_2max_) after endurance training (Timmons et al., 2010) and muscle fascicle length (Stebbings et al., 2018). An earlier study also revealed that the presence of at least one T allele enhanced the probability of reaching high performance levels in sports based on sprint and power, suggesting a possible genetic predisposition for power-based activities (Leońska-Duniec et al., 2022). Nevertheless, studies on this polymorphism are scarce and the obtained results are inconclusive. Understanding the genetic contributions to a sport-related phenotype could lead to individualized exercise prescription and training programs to optimize athletic development (Miyamoto-Mikami et al., 2018).

Based on the above-mentioned roles of the titin filament, in terms of developing muscle properties, we aimed to determine whether the *TTN* C > T polymorphism (rs10497520) moderated training-induced changes in selected variables of aerobic and anaerobic capacity. Accordingly, genotype distribution was measured in Caucasian females completing a 12-week training program to investigate potential interactions between the genotype and physiological adaptations. Aerobic capacity, as measured with a VO_2max_ test, as well as anaerobic capacity, as evaluated by the Wingate anaerobic test, were examined before and after the training intervention.

## Methods

### Participants

The study cohort comprised 156 healthy unrelated females (age: 21 ± 1 years). The criteria for study inclusion were as follows: a low level of self-reported physical activity, Caucasian origin, nonsmokers, not using any medications or supplements, and no apparent metabolic, musculoskeletal or neuromuscular diseases.

Each study participant received a dietary program, which included a recommendation of foods and drinks to achieve a balanced diet. On average, the following daily macronutrient ratio was suggested: 45–65% of carbohydrates, 20–35% of fat, and 10–20% of protein. Additionally, females were recommended to reduce cholesterol in diet to reach the level below 300 mg a day, with a minimal amount of fiber consumption of 25 g.

Before implementation of the training program, the maximum heart rate (HR_max_, %) of each participant was assessed using a continuous graded exercise test on an electronically-braked cycle ergometer (Oxycon Pro, Erich JAEGER GmbH, Hoechberg, Germany). The training intervention was preceded by a short familiarization period (3 sessions × 30 min at ~50% of HR_max_). All training sessions included a warm-up (10 min), the main exercise of high or low impact (43 min), and a cool-down phase consisting of low intensity exercises with stretching (7 min). A combination of exercises, based on running, skipping, lunges, knee bends, and hopping, formed the basis of the main training stimulus. The individual heart rate (HR) was registered with wearable HR monitors to supervise the intensity of exercise. Participants were informed to maintain earlier indicated HR ranges or a relative value of HR_max_. The 12-week training program was divided into four 3-week stages, each with 9 sessions. Each subsequent stage was characterized by increasing HR_max_ (from 50–60% to 65–80%) and beats per minute (BPM) (from 135–140 to 145–160): (1) HR_max_ about 50%–60%, tempo 135–140 BPM; (2) HR_max_ 60%–70%, tempo 140–152 BPM; (3) HR_max_ 65%−75%, tempo 145–158 BPM; and (4) HR_max_ 65%−80%, tempo 145–160 BPM.

### Tests

Before and after the 12-week training program, each participant completed VO_2max_ and Wingate anaerobic tests. To determine VO_2max_, participants performed a graded exercise test on an electronically-braked cycle ergometer (Oxycon Pro, Erich JAEGER GmbH, Hoechberg, Germany). Testing started with 5 min of constant pedaling at a frequency of 60 revolutions per minute (RPM) with a relative load of 1.5 W/kg^−1^. The workload increased by 15 W every minute until volitional exhaustion. The test was terminated when pedaling frequency declined by 10% (i.e., below 54 RPM). The highest oxygen uptake value maintained for a period of 15 s was considered VO_2max_. Anaerobic threshold values were also obtained using the V-slope method.

The Wingate anaerobic test was performed on a friction-braked cycle ergometer (Monark - model 864, Monark, Sweden). Saddle height was adjusted individually to each participant. Toe clips with straps were used to prevent feet slippage off the pedals. Before testing, a 5-min warm-up was conducted at 60 RPM with a workload of 50 W, followed by a 5-min recovery period. The 30-s Wingate test required participants to cycle as fast as possible against a constant resistance of 7.5% of body mass. Participants received verbal support to maintain maximal effort throughout the 30-s test. The total number of flywheel revolutions was counted (via a photocell) and data recorded by a computer-interfaced analog were transferred to a digital converter. Power output was recorded in W at 5-s intervals. Peak power (in W) and anaerobic capacity (in W/kg^−1^) were calculated, as well as the fatigue index (FI), considered as the percentage decline in power output across the test. In addition, time to reach maximal power (Tr) and time to maintain maximal power (Tm) were recorded (Castañeda-Babarro, 2021).

### Genetic Analyses

DNA was extracted from the buccal cells with a GenElute Mammalian Genomic DNA Miniprep Kit (Sigma, Steinheim, Germany), according to the manufacturer’s procedures. All samples were genotyped in duplicate, using an allelic discrimination assay on a C1000 Touch Thermal Cycler (Bio-Rad, Feldkirchen, Germany) instrument with TaqMan® probes. To discriminate the *TTN* rs10497520 alleles, TaqMan® Pre-Designed SNP Genotyping Assays were used (Applied Biosystems, Waltham, MA, USA) (assay ID: C___1958912_10). The assays contained primers and fluorescently-labeled (FAM and VIC) probes to identify the *TTN* alleles.

### Statistical Analysis

The Hardy-Weinberg equilibrium (HWE) was tested by comparing the expected and observed frequency of genotypes using the Chi-square test with one degree of freedom. Normality of data was evaluated with visual inspection of histograms, the Kolmogorov-Smirnov test, and kurtosis and asymmetry measures. Additionally, we used a two-way factorial analysis of variance (ANOVA) with a genotype as a between-subject factor and training as a within-subject factor. The main effects of the genotype and training, as well as genotype × training interactions, were evaluated. Contrast analyses were performed when appropriate. All analyses were carried out using STATISTICA software (version 13, www.statsoft.com).

## Results

Genotype distribution was consistent with the Hardy-Weinberg’s law at the level of significance of 5% (Chi-square = 0.01478, *p* = 0.993). Except for work achieved at P_max_ (J/kg), FI, and HR_max_, all aerobic and anaerobic capacity variables improved significantly during the applied training program (Tables 1 and 2).

**Table 1:** *TTN* rs10497520 genotypes and Wingate test variables before and after training (two-way mixed ANOVA).

V	CC (n = 124) Pre Post		Δ [%]	CT + TT (n = 32) Pre Post		Δ [%]	Genotype	Training	G x T
Peak power (W/kg)	7.88 ± 0.86	8.27 ± 0.89	4.9	7.77 ± 0.82	8.23 ± 0.74	6.0	0.656	**<0.001**	0.500
Tr (s)	6.63 ± 2.44	5.60 ± 2.043	15.4	6.95 ± 2.093	5.76 ± 2.128	17.2	0.533	**<0.001**	0.693
Tm (s)	3.79 ± 1.42	4.29 ± 1.71	13.1	3.46 ± 1.26	4.28 ± 1.73	23.6	0.512	**<0.001**	0.327
Force-speed coefficient (W/s)	48.16 ± 16.19	53.27 ± 18.51	10.6	47.36 ± 18.27	53.26 ± 23.93	12.4	0.904	**<0.001**	0.775
AnC(J/kg)	189.04 ± 19.46	199.64 ± 19.77	5.6	184.18 ± 16.70	200.93 ± 15.61	9.1	0.622	**<0.001**	**0.003**
Work to get P_max_ (J/kg)	54.27 ± 13.50	53.86 ± 13.82	0.8	53.26 ± 12.48	53.82 ± 16.88	1.06	0.824	0.958	0.731
Work after get P_max_ (J/kg)	134.76 ± 22.91	145.79 ± 22.45	8.2	130.90 ± 20.47	147.10 ± 20.76	12.4	0.752	**<0.001**	0.153
FI (%)	19.60 ± 5.78	20.05 ± 5.49	2.3	20.17 ± 5.32	19.08 ± 4.49	5.4	0.836	0.481	0.097

Mean ± standard deviation; p values (ANOVA) for main effects (genotype and training) and genotype x training interaction; bold p values: statistically significant differences (p < 0.05); V: variables; G x T: Genotype x training interaction, Tr: time to reach P_max_; Tm: time of maintaining P_max_; P_max_: maximal power; AnC: anaerobic capacity; FI: fatigue index

**Table 2:** The *TTN* rs10497520 genotypes and VO_2max_ test variables before and after training (two-way mixed ANOVA).

V	CC (n = 124) Pre Post		Δ [%]	CT + TT (n = 32) Pre Post		Δ [%]	Genotype	Training	G x T
VO_2max_ (ml/kg/min)	33.8 ± 5.2	37.6 ± 6.2	11.1	33.7 ± 4.5	36.3 ± 5.3	7.5	0.447	**<0.001**	0.213
VO_2_/AT (mL/kg/min)	24.72 ± 4.39	27.84 ± 4.99	12.6	24.08 ± 3.71	26.94 ± 4.07	11.9	0.292	**<0.001**	0.803
HR_max_ (beat/min)	186.95 ± 9.26	187.57 ± 8.31	0.3	185.88 ± 9.85	182.75 ± 12.91	1.7	0.067	0.192	0.051
HR/AT (beat/min)	163.23 ± 13.04	165.18 ± 10.56	1.2	163.75 ± 12.31	166.13 ± 10.74	1.5	0.727	**0.039**	0.836
VE_max_ (L/min)	71.56 ± 17.83	79.79 ± 18.39	11.5	70.93 ± 18.65	75.67 ± 18.68	6.7	0.473	**<0.001**	0.237
VE/AT (L/min)	38.22 ± 9.03	44.52 ± 10.79	16.5	37.38 ± 7.09	42.47 ± 10.83	13.6	0.380	**<0.001**	0.558

Mean ± standard deviation; p values (ANOVA) for main effects (genotype and training) and genotype x training interaction; bold p values: statistically significant differences (p < 0.05); V: variables; G x T: Genotype x training interaction; VO_2max_: maximum oxygen uptake; VO_2_/AT: oxygen uptake at the anaerobic threshold; HR_max_: maximum heart rate; HR/AT: heart rate at the anaerobic threshold; VE_max_: maximum minute ventilation; VE/AT: minute ventilation at the anaerobic threshold

Two-way ANOVA did not reveal any significant effects of the genotype on the Wingate test (Table 1) or VO_2max_ variables before and after training (Table 2), despite phenotypic differences in the rate of change induced by training reaching up to 10% (Table 1) in favor of the group with CT + TT genotypes. However, training and genotype interaction for anaerobic capacity (AnC) across the Wingate anaerobic test was significant (F1,154 = 8.95, *p* = 0.003, Table 1). CT and TT genotypes were associated with a larger increase (9.1% vs. 5.6%) in the AnC variable in response to training compared to the CC genotype (95%CI 2.09–10.20).

## Discussion

The major theoretical and practical foundations of physiologic adaptations to regular physical activity are well defined. In recent decades, important advancements in analytical laboratory techniques regarding biochemistry and molecular biology, among others, have led to significant advancements in sport and exercise science. Whether in disease prevention or sport skill enhancement, the genetic basis of exercise physiology has provided a framework to advance knowledge of molecular mechanisms involved in adaptation to regular physical activity (Gomes et al., 2020; Ipekoglu et al., 2023; Leońska-Duniec et al., 2016). Understanding the role of genetic determinants will likely improve the characterization, assessment, and prescription of exercise (at the individual level) to make training programs more efficient, safer, and to achieve better performance and greater health gains (Switala and Leonska-Duniec, 2021).

The most important finding of the present study was a significant genotype and training interaction for AnC during the Wingate test (*p* = 0.003). Specifically, carriers of the CT and TT genotypes demonstrated a larger increase in the total amount of work performed following the training intervention. In addition, we showed that aerobic training improved all the Wingate test variables in the CT + TT genotype group by 10%. This observation suggests that these genotypes may enhance changes in anaerobic power and capacity induced by regular training. Consequently, CC homozygotes would require a different training stimulus than T allele carriers to obtain comparable training gains. It should be noticed, however, that even though carrying specific *TTN* alleles may provide some advantage to achieving elite athlete status, it does not prevent athletes from achieving this status if they are absent (Petr et al., 2019). The Wingate test offers an effective tool for measuring both muscular power and anaerobic capacity, which engages the anaerobic energy pathways (e.g., stored ATP, creatine phosphate system) (Krishnan et al., 2017). Anaerobic power is a critical component of sporting success, especially for performance in events where short-term explosive efforts lasting up to approximately 8 s are required. Therefore, in this context, the Wingate test is a valid and reliable tool for coaches, athletes, and scientists (Krishnan et al., 2017; Zupan et al., 2009).

To our knowledge, few data are available on the functional relevance of polymorphisms in the *TTN* gene. Firstly, in the HERITAGE Family Study, a genome-wide linkage scan for endurance training-induced changes in submaximal exercise stroke volume (ΔSV50) was undertaken. This study consisted of 483 participants and revealed 2q31-q32 and 10p11.2 chromosomal regions with evidence of an association among white families. Furthermore, Rankinen et al. (2004) found *TTN* to be a very promising candidate gene for human variation in ΔSV50, both in a sedentary state and training responsivity. Those authors also observed that titin was the main contributor to cardiomyocyte elasticity and a key regulator of the Frank-Starling mechanism, thereby emphasizing that statistically and biologically this was the best candidate for cardiac adaptation to endurance training. Further evidence (from HERITAGE Family Study) was provided by Timmons et al. (2010) who combined RNA expression profiling with analysis of single nucleotide polymorphisms (SNPs) associated with increases in VO_2max_ among 473 Caucasian men. This experiment showed that the *TTN* rs10497520 polymorphism was one of the 11 SNPs which explained 23% of the variance in training gains in VO_2max_. Unfortunately, our study did not confirm this association between the *TTN* polymorphic site and change in VO_2max_ variables induced by regular physical exercise. Classical twin studies have estimated the heritability of peak oxygen uptake, cardiac mass, and structure to be between 40% and 70%, with broad diversity in the sample size, ethnic variation, physical activity, diet, age, sex, as well as different phenotype measurement procedures, leading to inconsistent results (Costa et al., 2012).

In another study, Stebbings and co-workers (2018) analyzed the *TTN* polymorphism associations with the length of skeletal muscle fascicle in recreationally active Caucasian males (n = 137) and personal best times in marathon runners (n = 141). Although no between-group differences in genotype frequency emerged, they showed that marathon personal best time was significantly lower in T allele carriers compared to the CC homozygotes. Additionally, in the recreationally active group, they found that muscle fascicle length of the vastus lateralis was 10.4% longer in the CC homozygotes than in the CT heterozygotes. The T allele carriers were suggested to possess shorter vastus lateralis fascicles which need fewer energy to produce a certain force and thus, might favor marathon-running performance. Conversely, CC genotype carriers might have longer vastus lateralis fascicles which, in turn, might benefit sprint-running performance (Stebbings et al., 2018). Surprisingly, a previous study focused on whole-genome sequencing combined with association testing of chosen variants within the *TTN* gene with sport-related traits, showed that the T allele was linked to sprint and power performance. Specifically, the T allele carriers were more than two times more likely to become elite athletes in sports where the anaerobic energy system predominates (Leońska-Duniec et al., 2022).

This study represents the next logical step in understanding the relationship between polymorphisms located in the *TTN* gene and various aspects of sports performance and adaptation. We provided evidence that individuals with CT and TT genotypes demonstrated significantly greater total work during Wingate testing. Arguably, this result implies that these genotypes are predisposed to achieving greater adaptive changes in anaerobic power, which is critical to success in sprint- and power-based sports. Therefore, our study confirms earlier results (Leońska-Duniec et al., 2022) and indicates that carrying CT and TT genotypes is beneficial in sports where the anaerobic exercise metabolism predominates.

Literature examining the genetic contribution to short-duration motor tasks is quite significant. Heritability has been estimated at the level of 30–90% (Costa et al., 2012) and the studied *TTN* variant seems to be one of the SNPs which explains the variance in training-induced changes in anaerobic power and capacity. The *TTN* polymorphisms may take part in changeability within expression of the titin isoform in skeletal and cardiac muscle tissue (Stebbings et al., 2018). Titin length is related directly to sarcomere properties, such as elasticity, stiffness, and elongation (Freundt and Linke, 2019). Variations in the I-band region, in particular the immunoglobulin (Ig) areas and the PEVEK region (being abundant in proline, glutamic acid, valine, and lysine), affect the length of titin filaments. It has been established that shorter isoforms are more stiff, while the longer are more elastic (Chauveau et al., 2014; Freiburg et al., 2000; Labeit and Kolmerer, 1995). As such, changes in the *TTN* gene sequence may modify the production of different length filaments, thereby affecting contractile characteristics. In addition, some SNPs such as the missense rs10497520 polymorphism which is localized in the fragment of the *TTN* gene encoding the myofilament Z-disc region may affect regular length titin isoforms, but are weakly connected to the Z-disc, with downstream effects on sarcomere mechanical stability. Thus, molecular changes in this region may be favorable or unfavorable for expressing muscular performance (Leońska-Duniec and Maciejewska-Skrendo, 2021; Stebbings et al., 2018).

## Conclusions

Our study provides further evidence of an association between the *TTN* rs10497520 polymorphism and training-induced anaerobic adaptations. We showed that harboring at least one of the rs10497520 T allele improved the Wingate test variables, in particular AnC (*p* = 0.003), in Caucasian women exposed to a 12-week training program. The obtained results suggest that the CT and TT genotypes may enhance changes in anaerobic power and anaerobic capacity induced by regular training. We also suggest that T allele carriers may possess a metabolic adaptive advantage towards anaerobic metabolism.. Consequently, CC homozygotes would require a different training stimulus from the T allele carriers to obtain comparable training gains. In light of this evidence, the *TTN* gene appears to offer a promising genetic marker for regulating sports performance and adaptation, which may also underlie individual differences in achieving success in sprint-power events. Further research is needed to verify the role of this polymorphism and others localized in the *TTN* gene, as well as their linkages to humans’ health, physical activity, athletes’ status, and injury risk
